# Supplementary figures and images for: Illumina RNA and SMRT Sequencing Reveals the Mechanism of Uptake and Transformation of Selenium Nanoparticles in Soybean Seedlings
Source: Plants (Basel). 2023 Feb 9;12(4):789. doi: 10.3390/plants12040789 (PMC9966555; doi:10.3390/plants12040789)

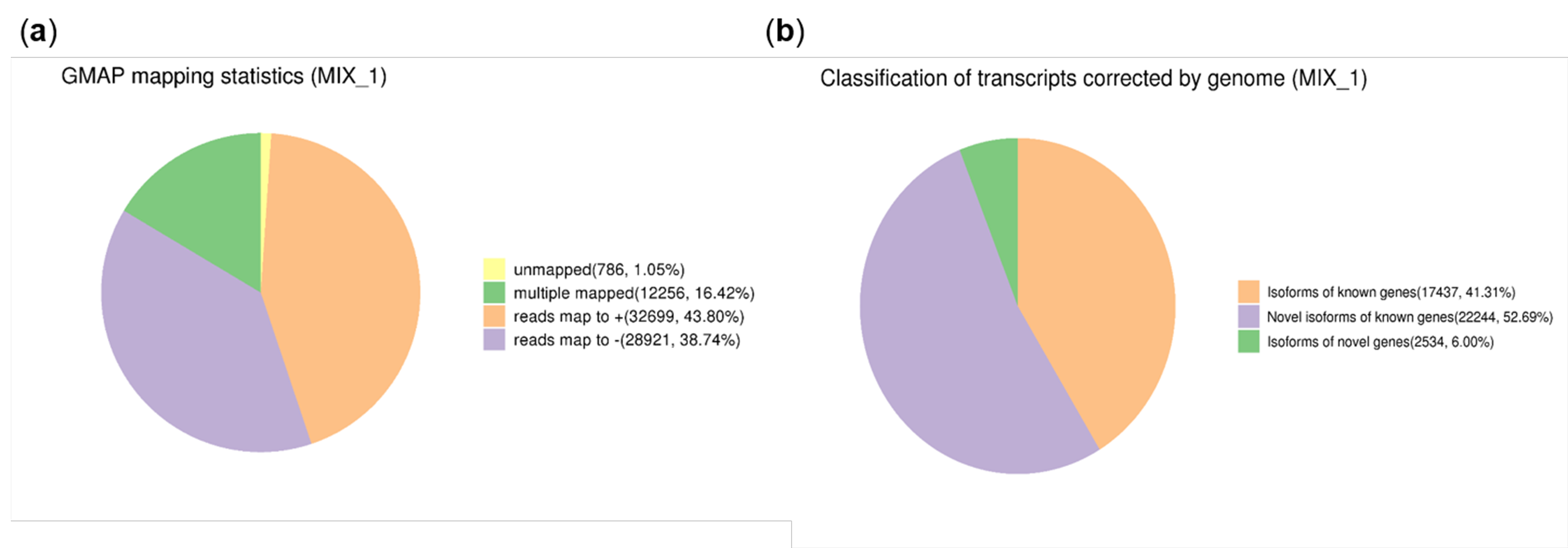

Supplement: Supplementary file 1 [file plants-12-00789-s001.zip › plants-2194058-supplementary/Supplementary files-R1/Supplementary Figure S1.tif]

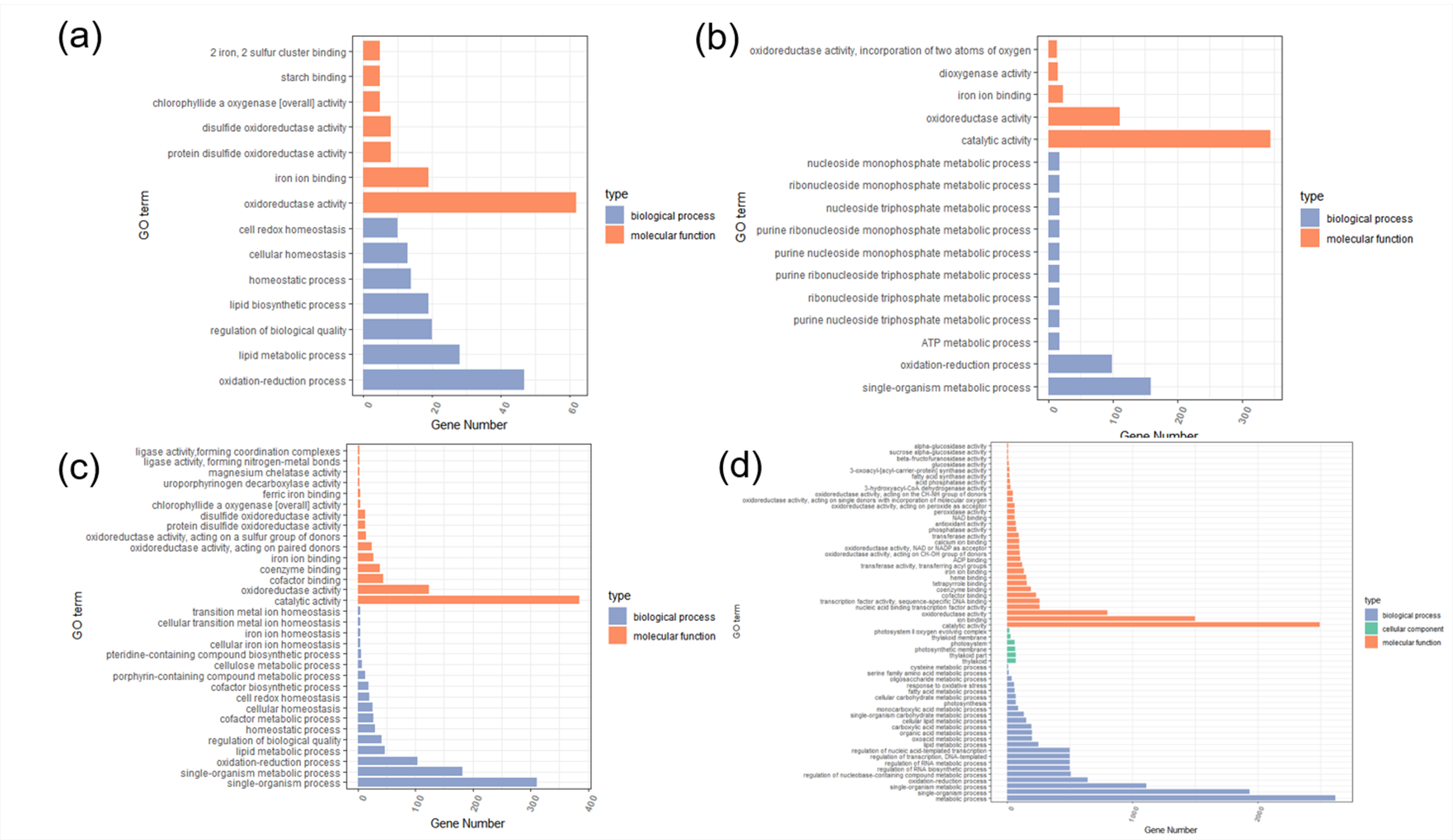

Supplement: Supplementary file 1 [file plants-12-00789-s001.zip › plants-2194058-supplementary/Supplementary files-R1/Supplementary Figure S2.tif]

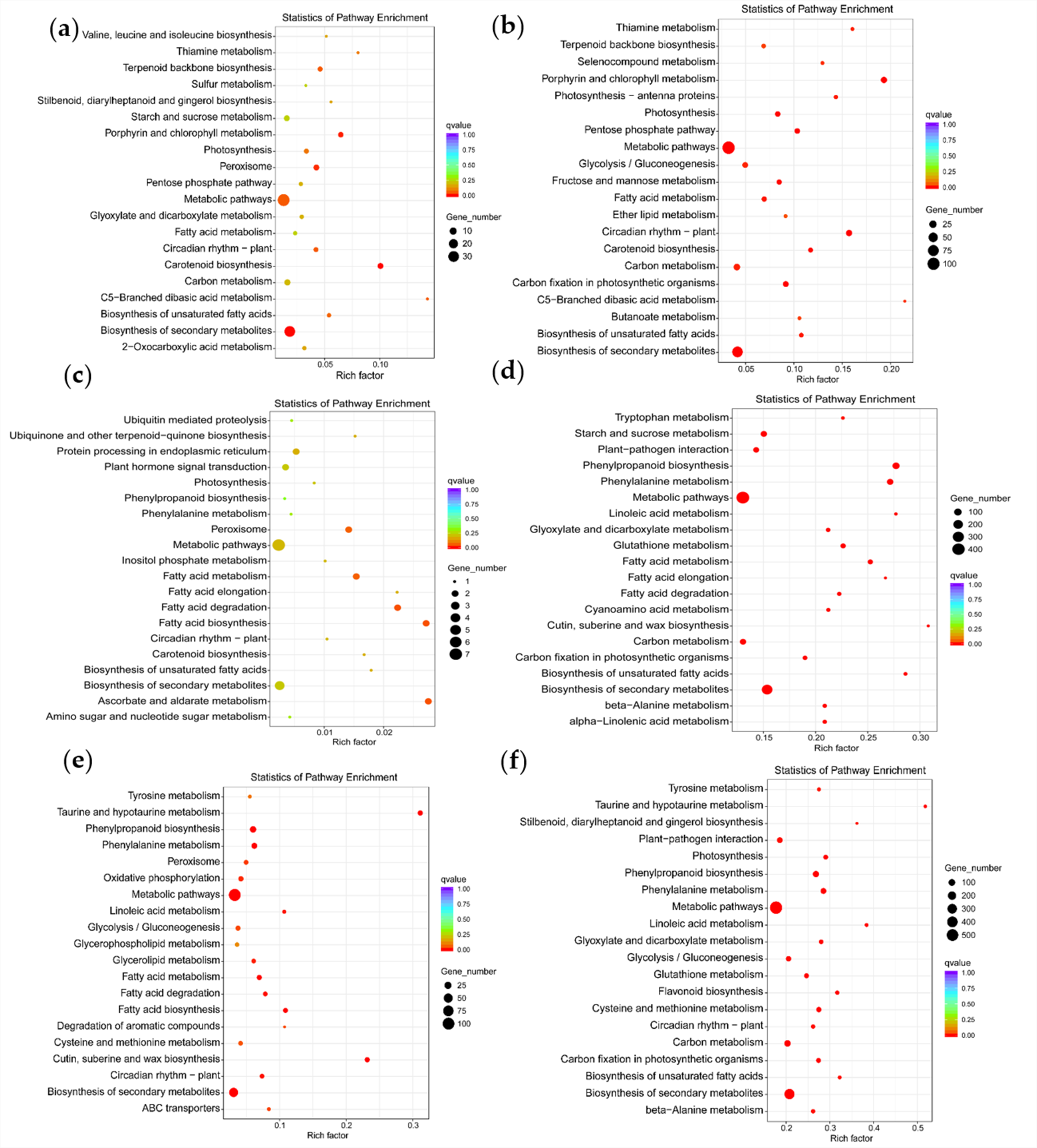

Supplement: Supplementary file 1 [file plants-12-00789-s001.zip › plants-2194058-supplementary/Supplementary files-R1/Supplementary Figure S3.tif]

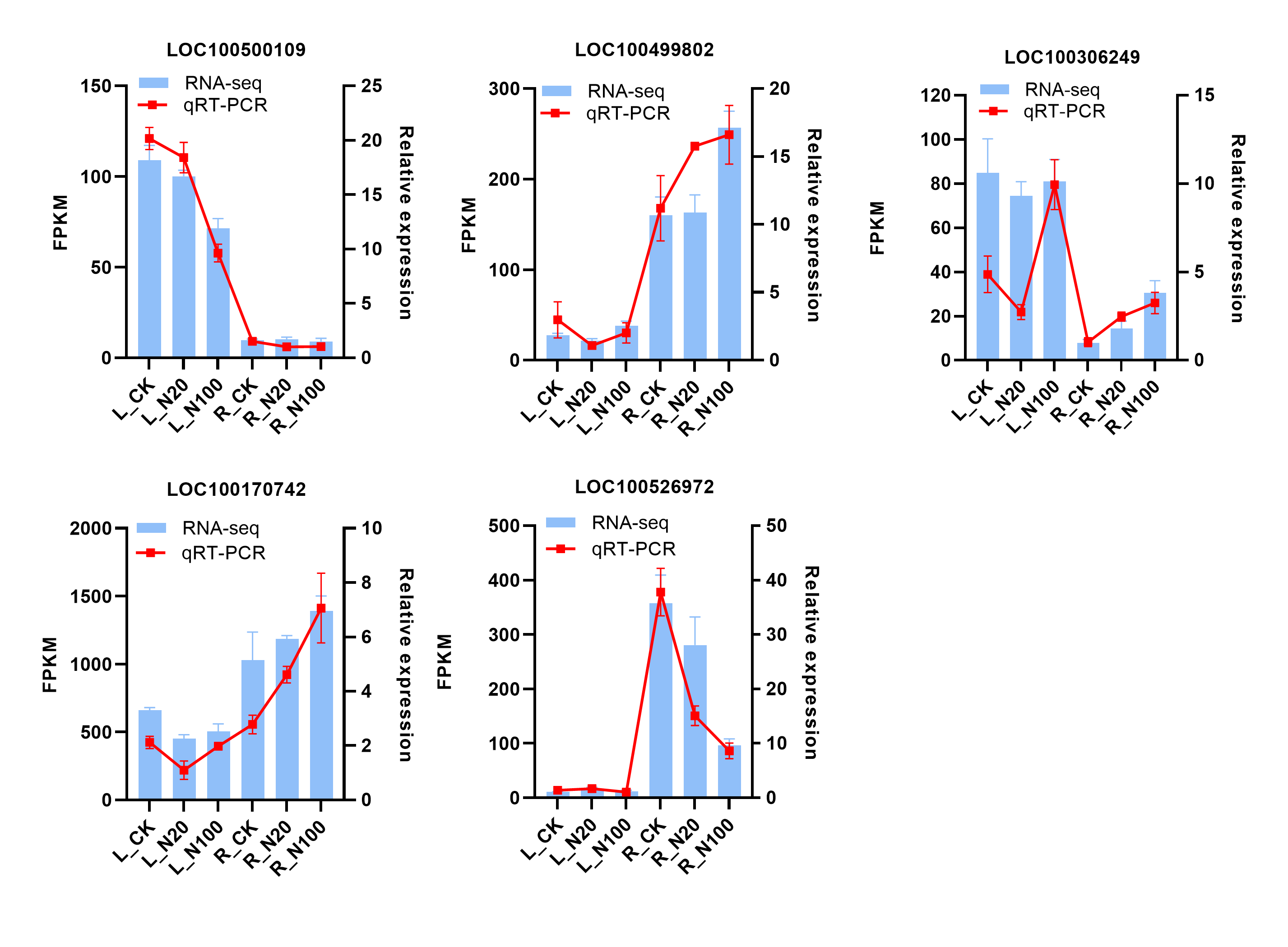

Supplement: Supplementary file 1 [file plants-12-00789-s001.zip › plants-2194058-supplementary/Supplementary files-R1/Supplementary Figure S4.tif]
